# Gender Differences in the Association between Socioeconomic Status and Subclinical Atherosclerosis

**DOI:** 10.1371/journal.pone.0080195

**Published:** 2013-11-25

**Authors:** Olivier Grimaud, Annabelle Lapostolle, Claudine Berr, Catherine Helmer, Carole Dufouil, Wahida Kihal, Annick Alpérovitch, Pierre Chauvin

**Affiliations:** 1 French School of Public Health, Rennes, France; 2 Institut National de la Santé et de la Recherche Médicale U707, Paris, France; 3 Institut National de la Santé et de la Recherche Médicale, U1061, Montpellier, France; 4 Institut National de la Santé et de la Recherche Médicale, U897, Bordeaux, France; 5 Université Bordeaux Segalen, Bordeaux, France; 6 Institut National de la Santé et de la Recherche Médicale 708, Paris, France; INRCA, Italy

## Abstract

**Objectives:**

This study explored the pattern of associations between socioeconomic status (SES) and atherosclerosis progression (as indicated by carotid intima media thickness, CIMT) across gender.

**Design:**

Cross-sectional analysis of a sample of 5474 older persons (mean age 73 years) recruited between 1999 and 2001 in the 3C study (France). We fitted linear regression models including neighborhood SES, individual SES and cardiovascular risk factors.

**Results:**

CIMT was on average 24 µm higher in men (95% CI: 17 to 31). Neighborhood SES was inversely associated with CIMT in women only (highest versus lowest tertiles: −12.2 µm, 95%CI −22 to −2.4). This association persisted when individual SES and risk factors were accounted for. High individual education was associated with lower CIMT in men (−21.4 µm 95%CI −37.5 to −5.3) whereas high professional status was linked to lower CIMT among women (−15.7 µm 95%CI: −29.2 to −2.2). Adjustment for cardiovascular risk factors resulted in a slightly more pronounced reduction of the individual SES-CIMT association observed in men than in women.

**Conclusion:**

In this sample, neighborhood and individual SES displayed different patterns of associations with subclinical atherosclerosis across gender. This suggests that the causal pathways leading to SES variations in atherosclerosis may differ among men and women.

## Introduction

There is good evidence that individual socioeconomic status (SES) is associated with cardiovascular risk. The gradient of increasing risk when SES decreases has long been demonstrated in studies measuring cardiovascular events such as myocardial infarction [1] and stroke [2]. There is also evidence that contextual factors, for instance the SES of the area of residence, are associated with cardiovascular health, which is worsened in socially deprived neighborhoods after controlling for individual SES [3]. More recently, studies have explored the link between SES and subclinical atherosclerosis as measured by the carotid intima media thickness (CIMT). CIMT measurement by ultrasound is relatively simple, non-invasive and correlates well with histology [4]. In their meta-analysis, Lorenz et al showed that a 0.10 mm increase of the common carotid intima media thickness corresponds to a 1.15 relative risk of developing myocardial infarction (95% CI, 1.12 to 1.17), and 1.18 relative risk for stroke (95% CI 1.16 to 1.21) [5]. Previous studies have shown that low education, low income, or manual occupation are associated with thicker carotid arterial wall [6]–[11] or with faster progression of carotid wall thickness [12]. Neighborhood SES measured either instantaneously [13]–[16] or over the lifecourse [17], [18] also shows an inverse association with CIMT.

To date the few studies which have investigated the link between SES and CIMT have been conducted in the USA and in Northern Europe. Since evidence in this domain of research only rely on observational studies, findings from different epidemiological settings help strengthening the case for a causal link. Our first aim was therefore to examine whether similar associations existed in France, a country where cardiovascular diseases (CVD) represent a less prominent part of the disease burden, [19] but is not void of significant health inequalities [20]. Cardiovascular diseases develop 7 to 10 years later in women, most probably as a consequence of exposure to endogenous estrogens and lower prevalence of risk factors [21]. Since results from earlier studies suggested that the influence of SES on the development of subclinical atherosclerosis differs in men and women [11], [14], [17] we also examined the pattern of the SES-CIMT associations across gender.

## Methods

The 3C study is an ongoing longitudinal study on vascular risk factors and dementia. For the present cross sectional analysis we used information collected at baseline. Full description of the study design has been reported elsewhere [22]. Briefly, between 1999 and 2001, non-institutionalized persons aged 65 years and over were recruited from the electoral rolls of Bordeaux, Dijon and Montpellier. Eligible inhabitants were invited to participate and the overall acceptance rate was 37% giving a total number of 9294. Each participant signed an informed consent. The study protocol received approval from the Ethical Committee of the University Hospital of Kremlin-Bicêtre. Ultrasound examination of the carotid arteries was proposed only to participants less than 85 who were able to come to the examination centers. For costs constraints, measurements could not be performed in the last 4 months of the baseline phase of the study. Overall, valid ultrasound measurements were available for 69.5% of the participants (n = 6462).

### Measurement of carotid intima media thickness

As described elsewhere [23], [24] ultrasound examinations followed a common protocol and all results were read centrally at the reference center (Hôpital Broussais, Paris). Each study center used a B-mode system (Ultramark 9 High Definition Imaging) with a 5 to 10-MHz sounding. Carotid intima media thickness (CIMT) was measured at a plaque free site along a 10-mm-long segment of the far wall of right and left common carotid arteries 2 to 3 cm proximal to the bifurcation. It was measured as the distance between the lumen-intima interface and the media-adventitia interface using an automated edge detection algorithm. On average, 75 measurements were automatically performed on each side. The CIMT used in the analysis corresponds to the mean of the right and left mean values. A reproducibility study was conducted on 114 subjects who underwent 2 ultrasound examinations performed blindly by 2 different sonographers. The results showed a mean absolute difference of 60 µm and a correlation coefficient of 0.71 between repeated examinations which is comparable to figures found in other large community studies[25], [26].

### Individual and neighborhood socioeconomic variables

Education level was collected in 6 categories ranging from no diploma to university degree that we grouped in 3 classes: less than 6 years, 6 to 11 years and ≥12 years of schooling. Professional status was also grouped in 3 categories: high (managers, professionals, technicians and associate professionals) intermediate (clerical, service, shop and market sale workers, armed forces) and low (all other professions including housewife). Household income was initially collected in 4 categories. Since only 272 participants (5%) declared monthly income of less than 750€ per month, these were grouped to the next level of income in order to obtain 3 groups of comparable size (<1500€, 1500-2250€, >2250€).

Postal addresses were geocoded in order to match participants to their neighborhood of residence. As done in other studies [27], [28], we used IRIS (for “Ilots Regroupés pour l’Information Statistiques”) as proxy for neighborhoods. IRIS is the smallest census aggregation level used by the French national statistical office to disseminate information. It corresponds to homogenous geographical areas with borders that follow main roads or other physical features such as rivers or railways. At the time of the 1999 population census in France, the average population size of IRISs was 2400. Because Bordeaux, Dijon and Montpellier represent socially and culturally contrasted urban settings and displayed significantly different values of mean CIMT, we tried to identify a common neighborhood SES indicators which fulfilled the two following properties: 1) showed a large range of values between IRISs in each center so as to increase discrimination power, 2) displayed comparable distributions across centers (as reflected by medians and interquartile values) so as to limit confounding. We used the 1999 census and the 2001 “household tax income” data files provided by the French National Office of Statistics to derive unemployment rate, proportion of manual workers, median annual income, proportion of adults with secondary education and the Townsend deprivation score. The proportion of adults with secondary education proved to best fit our two criteria and was therefore selected as neighborhood SES indicator.

### Risk factors variables

Measurements of height, weight, systolic and diastolic blood pressure, were performed during clinical examinations. One laboratory carried out measurements of all biological parameters, including fasting glycaemia and lipid levels. We entered diabetes as a 3 categories variable (glycaemia<6.1 mmol; 6.1≤glycaemia≤7 mmol/l; glycaemia>7 mmol/l or treatment), hypercholesterolemia as a binary variable (serum total cholesterol > 6.2 mmol/l, or treatment), systolic blood pressure in five categories (quintiles) and added a variable indicating use of antihypertensive treatment. Smoking status was included as former, current or none, alcohol consumption in 4 categories (abstinent, <10, <20 or ≥20 gr/day), and BMI (weight/height^2^) as a continuous variable.

### Statistical analysis

We first examined the distribution of individual SES variables, risks factors and mean CIMT. We used the Chi^2^ test and the Kruskall Wallis test to determine statistical significance of the differences across gender. We analyzed men and women separately and tested for gender SES interactions in models including all participants when results suggested differing associations across genders. Since multilevel models showed little evidence of clustering (intraclass correlation lower than 1%) we estimated parameters using single level multiple linear regression models. All models were adjusted for age and center. We started by exploring associations between CIMT and neighborhood SES categorized in tertiles. We then added individual SES variables in order to estimate their own associations with CIMT and their influence on the neighborhood SES-CIMT relationship. To better ascertain the influence of income, we fitted models restricted on the subsample of participants living on their own for whom household income therefore does not depend of household composition. In the final stage of the analysis we checked whether the associations between SES and CIMT were modified when accounting for cardiovascular risk factors. All analyses were performed with Stata v10.2 (StataCorp LP, TX, USA).

## Results

Valid ultrasound measures were available for 6462 participants. We excluded those having a self-reported history of cardiovascular disease (n = 847) and those who could not be matched to their neighborhood of residence (n = 110). Because estimation of the variance at the neighborhood level requires the presence of at least two participants in a given neighborhood, a further 31 “isolated” records were excluded. The final study sample consists thus of 5474 individuals (2007 men and 3467 women) with mean age 73 years, distributed in 242 neighborhoods. Mean CIMT was highest in Bordeaux (749 µm), intermediate in Montpellier (712 µm) and lowest in Dijon (695 µm, p<10^−3^).

Information on individual SES was missing for 324 (5.9%) participants, mostly because of missing household income (n = 305), who therefore could not be included in some models. Participants with missing individual SES were more often women (77% versus 62% among others, p<10^−3^) and older (mean difference +1.6 year, p<10^−3^) but had similar CIMT.

Men and women differed markedly according to socioeconomic status and risk factors profile (table 1). Men were more educated and had higher professional status. Nearly twice as many men than women declared household income in the highest category but this should be seen in light of the fact that women were more often living on their own (48.3 versus 13.5%). Smoking and alcohol drinking were much more frequent among men and overall, with the exception of hypercholesterolemia, their biological risk factors profile was less favorable. Men’s carotid wall was on average 24 µm (95%CI: 17 to 31) thicker than that of women.

**Table 1:** Means and percent distributions of socioeconomic status indicators and cardiovascular risk factors for the entire sample and by gender.

	Entire sample	Men	Women	*p* *
Number of participants	5 474	2 007	3 467	
Age (mean (sd) in years)	73.3 (4.9)	73.2 (4.9)	73.4 (4.8)	*0.30*
Living alone (%)	35.5	13.5	48.3	*<10^−3^*
Participant socioeconomic status (%)				
Education				*<10^−3^*
<6 years	31.2	26.8	33.8	
6−11 years	30.3	26.5	32.5	
≥12 years of schooling	38.5	46.7	33.7	
Professional status				*<10^−3^*
Low	38.8	32.6	42.4	
Intermediate	40.6	31.9	45.6	
High	20.6	35.4	12.0	
Household monthly income				*<10^−3^*
Low (<1 500€)	34.9	19.4	44.1	
Medium (1 500 to 2250€)	29.3	31.1	28.3	
High (>2 250€)	35.8	49.5	27.6	
Neighborhood socioeconomic status				
Number of neighborhoods	242			
Proportion of adults with secondary education in the neighborhoods (mean (sd))	53 (15)			
Behavioral risk factors				
Smoking (% former or current)	36.7	67.8	18.8	*<10^−3^*
Mean (sd) alcohol consumption (gr/day)	12.9 (14.7)	21.8 (18.2)	7.8 (8.9)	*<10^−3^*
Mean (sd) body mass index (kg/m^2^)	25.5 (4.0)	26.1 (3.4)	25.1 (4.2)	*<10^−3^*
Biological risk factors				
Mean (sd)systolic blood pressure (mm Hg)	145 (21)	150 (21)	142 (21)	*<10^−3^*
Receiving Anti-hypertensive therapy (%)	43.5	42.1	44.3	*0.11*
Diabetes (%)	8.5	11.8	6.6	*<10^−3^*
Hypercholesterolaemia (%)	55.0	41.7	62.7	*<10^−3^*
Carotid intima-media thickness				
Mean (sd) inµm	711 (120)	727 (129)	702 (113)	*<10^−3^*

3C study (France, 1999−2001).

* p-value for differences between gender (Kruskall-Wallis test for continuous variables and Chi^2^ test for categorical variables).

Neighborhood SES showed a significant association with CIMT in women (table 2). Among them, each increase in the tertile of neighborhood education corresponded to a decrease of approximately 6 µm of CIMT (linear trend in model 1, p = 0.02). This association persisted when individual SES (model 2) and risk factors (model 3) were accounted for. In men, although CIMT decreased with increasing level of neighborhood in model 1, this failed to reach statistical significance (linear trend, p = 0.30). Adjustment for individual SES and risk factors tended to attenuate the association. No significant gender-neighborhood SES interaction existed in model including both men and women.

**Table 2:** Associations between socioeconomic status and carotid intima media thickness. Values are differences in carotid intima media thickness measured inµm (95% CI) 3C study (France, 1999−2001).

		Men			Women	
	Model 1neighborhood SES(N = 2007)	Model 2model 1 + individual SES(N = 1933)	Model 3model 2 +risk factors^a^(N = 1933)	Model 1neighborhood SES(N = 3467)	Model 2model 1 + individual SES (N = 3217)	Model 3model 2 +risk factors^a^ (N = 3217)
Neighborhood SES^b^						
1st tertile (reference)	0	0	0	0	0	0
2nd tertile	−4.5	−5.8	−2.9	−7.1	−5.7	−4.5
	(−18.0; 9.0)	(−19.8; 8.1)	(−16.8; 10.9)	(−16.1; 1.8)	(−15.0; 3.9)	(−13.8; 5.0)
3rd tertile	−7.6	−5.9	−3.5	−12.2 *	−12.4 *	−10.8 *
	(−22.1; 6.8)	(−21.2; 9.5)	(−18.7; 11.7)	(−22.0; −2.4)	(−22.8; −2.0)	(−21.1; −0.4)
Individual education						
< 6 years (reference)		0	0		0	0
6−11 years		1.4	5.1		−7.9	−6.8
		(−14.1; 17.0)	(−10.4; 20.5)		(−17.7; 1.8)	(−16.6; 2.9)
≥12 years		−21.4 **	−16.9*		−2.9	−0.7
		(−37.5; −5.3)	(−32.9; −0.9)		(−13.7; 7.9)	(−11.6; 10.1)
Professional status						
Low (reference)		0	0		0	0
Intermediate		−0.3	3		−10.8*	−9.8*
		(−14.7; 14.1)	(−11.3; 17.2)		(−19.4; −2.1)	(−18.3; −1.2)
High		9.3	9.6		−15.7*	−13.3
		(−6.7; 25.3)	(−6.3; 25.4)		(−29.2; −2.2)	(−26.8; 0.1)
Household income						
Low (reference)		0	0		0	0
Medium		3.2	2.5		−0.7	1.3
		(−13.1; 19.6)	(−13.7; 18.7)		(−10.0; 8.6)	(−8.0; 10.6)
High		12.2	12.2		3.8	6.2
		(−4.6; 29.1)	(−4.5; 28.9)		(−6.4; 14.0)	(−4.0; 16.4)

* p<0.05 **p<0.01.

All models are adjusted for age and center.

a smoking; alcohol intake; body mass index; systolic BP; antihypertensive treatment; diabetes; hypercholesterolemia.

b Socioeconomic status of the neighborhood measured as the proportion of adults with secondary education.

The pattern of individual SES and CIMT associations also varied across gender. In men, no difference in CIMT was noticeable between the first two levels of education. However, compared to those with less than 6 years of schooling, those with 12 years or more had lower CIMT (−21.4 µm, p = 0.009). Adjustment for risk factors (model 3) reduced this association by 21%. Results regarding individual income in men were suggestive of a positive association with CIMT but this association failed to reach statistical significance (p for linear trend  =  0.13).

In contrast with men, only professional status was associated with CIMT among women (gender-professional status interaction: p<0.05). CIMT decreased as professional status increased and was significantly lower among women who had a non-manual profession (p for linear trend 0.007 in model 2 and 0.02 in model 3). This relationship was only slightly reduced when adjusting for risk factors. Only 498 women of our sample (14%) declared to be housewife and excluding them from the analysis tended to accentuate the association between non manual profession and CIMT (not shown). There was no discernible association between household income and CIMT, whether all women (model 2 and 3) or only those living alone were analyzed (not shown). Too few men were living on their own (n = 271) for us to derive a precise estimate of the income CIMT association in this subgroup.

## Discussion

We showed that the inverse association between socioeconomic status and subclinical atherosclerosis observed in the USA and northern Europe also applies to France, a country were cardiovascular diseases are less prevalent. Similar to others, we found that both individual and contextual SES factors contribute to this relationship. An interesting aspect of our results is the differential pattern of these associations across gender.

First, neighborhood SES showed a clearer negative association with CIMT in women than in men and this association was statistically significant only in the former. It is possible that our sample lacks statistical power in order to identify significant association in men. However, previous studies have shown a similar stronger effect among women of neighborhood SES on subclinical atherosclerosis [14], [17], incidence of coronary heart diseases [29] and self-rated health [30]. In adult life, women’s involvement in childcare and part-time work is likely to have resulted in them being more exposed than men to the physical and social aspects of their area of residence. Men and women may also differ in the ways they perceive their local environment and make use of health promoting services and facilities [30]. Neighborhoods that we classified as advantaged in our study on the basis of residents’ educational level are likely to offer more or higher quality of these services and facilities.

Our results also showed differences across gender with respect to the individual SES indicator associated with CIMT, namely education in men and professional status in women. Moreover, adjustment for cardiovascular risk factors suggests that they may explain part of the education-CIMT association in men, but less so of the profession-CIMT association in women. Evidence is accumulating regarding the role of psychosocial factors in the development of ischemic heart diseases [31]. For instance, work and marital stress were positively associated with faster progression of atherosclerosis in a prospective study of women hospitalized for an acute coronary event [32]. Results from animal studies indicate that the relation between social status and CVD may be sex-specific [33]. Specifically, social subordination may increase the risk of atherosclerosis in female but not in male monkeys, which somehow corroborates our findings regarding occupation. More generally, women may be more exposed and perhaps more susceptible to psychological stressors from their neighborhood, family and professional environments, whereas the contribution of adverse health behaviors might be more important in men of this generation. Taken together these results are compatible with the hypothesis that the causal pathways leading to social disparities in atherosclerosis differ across gender.

### Strengths and limitations

Strengths of our study include the large sample size, standardized measurements for CIMT and other co-variables, and availability of both individual and neighborhood SES. Assessment of the latter may suffer from measurement error however, since lifetime (as opposed to current) exposure to neighborhood deprivation is more closely linked to subclinical atherosclerosis [14], [17]. Also, we had no information on non-residential neighborhood exposures which have been shown to confound the association between health and residential neighborhood [34]. Our results may therefore underestimate the strength of neighborhood CIMT associations. Similarly, relying on current as opposed to lifetime exposure to risk factors may underestimate their contributions to social health inequalities [35]. The 3C study’s initial 37% acceptance rate raises the issue of selection bias, which would arise if acceptance was differentially linked to health across SES [36]. Assuming that higher SES individuals were more willing to take part to 3C despite lower health level, our results would again underestimate the “true” negative association between SES and CIMT. Finally, because of the cross-sectional design of our study, the associations we identified do not necessarily reflect causal relationships.

## Conclusion

In conclusion, this study adds to the body of evidence suggesting that both contextual and individual SES play a part in the development of subclinical atherosclerosis. The pattern of gender differences that we observed is consistent with the hypothesis that, at the time of data collection, the causal pathways leading to social inequalities in atherosclerosis differed across gender. Although a different mix of exposure to social and cardiovascular risk factors may now prevail, public health actions aiming at reducing these inequalities may still need adaptation to these differences.
